# Nitric Oxide, Oxidative Stress, and p66^Shc^ Interplay in Diabetic Endothelial Dysfunction

**DOI:** 10.1155/2014/193095

**Published:** 2014-03-05

**Authors:** Alessandra Magenta, Simona Greco, Maurizio C. Capogrossi, Carlo Gaetano, Fabio Martelli

**Affiliations:** ^1^Vascular Pathology Laboratory, Istituto Dermopatico dell'Immacolata-IRCCS, 00167 Rome, Italy; ^2^Molecular Cardiology Laboratory, Policlinico San Donato-IRCCS, San Donato Milanese, 20097 Milan, Italy; ^3^Division of Cardiovascular Epigenetics, Department of Cardiology, Internal Medicine Clinic III, Goethe University, 60590 Frankfurt am Main, Germany

## Abstract

Increased oxidative stress and reduced nitric oxide (NO) bioavailability play a causal role in endothelial cell dysfunction occurring in the vasculature of diabetic patients. In this review, we summarized the molecular mechanisms underpinning diabetic endothelial and vascular dysfunction. In particular, we focused our attention on the complex interplay existing among NO, reactive oxygen species (ROS), and one crucial regulator of intracellular ROS production, p66^Shc^ protein.

## 1. Introduction

Endothelial cells (ECs) synthesize and release different molecules that orchestrate metabolic, vascular, and cellular responses. Among them, nitric oxide (NO) is a key regulatory molecule of paramount importance for endothelial function and vascular tone relaxation [1, 2]. Notably, reduced endothelial cell nitric oxide synthase (eNOS) expression and/or NO bioavailability are associated with decreased EC survival and with endothelial dysfunction [3]. Indeed, a dysfunctional endothelium is not able to oppose vasoconstrictor stimuli, causing the increase of the arterial tone.

Reactive oxygen species (ROS), which include hydrogen peroxide (H_2_O_2_), superoxide anion (O_2_^−^), and hydroxyl radicals, also play a pivotal role in endothelial and vascular function as well as in vascular tone constriction [4]. ROS are generated as a consequence of aerobic metabolism and are produced by several cellular sources: plasma membrane NADPH oxidase (NOX), mitochondria, and different enzymes, such as several oxidases, peroxidases, cytochromes, mono- and dioxygenases, and uncoupled NOS.

The amount of ROS within the cell is finely modulated by enzymatic and nonenzymatic antioxidant defenses such as superoxide dismutases (SODs), catalase (CAT), glutathione peroxidase (GPx), and glutathione. Physiological ROS levels play an important role as second messengers within the intracellular signaling. Indeed, ROS can be actively generated and mediate physiological intracellular signalling as second messengers [5]. However, ROS production exacerbation or insufficient scavenging has been demonstrated to impair many biological processes including endothelial function in several pathological contexts.

A strict link exists between NOS activity and ROS production, since NOS uncoupling leads to the production of superoxide anion rather than NO. One of the major determinants of NOS uncoupling is the bioavailability of the cofactor tetrahydrobiopterin (BH_4_) [6]. ROS as well as peroxynitrite (ONOO^−^), another potent oxidant produced by the reaction of superoxide anion with NO, induce BH_4_ degradation leading to eNOS uncoupling and to a reduction of the amount of endothelium-derived NO that is required for vascular relaxation and EC survival and proliferation [7].

The cellular pathways induced by ROS increase are known to provoke growth arrest and senescence, as well as cell death, either by apoptosis or by necrosis, according to the level of oxidative stress experienced by the cell, its genotype, and a multitude of epigenetic changes [8, 9]. A pivotal role in ROS-induced apoptosis is played by the p66 isoform of ShcA protein (p66^Shc^), a fundamental regulator of mitochondrial ROS production by a variety of different stimuli [10]. Moreover, a fundamental role played by microRNAs is emerging [11, 12], indicating that noncoding RNAs play a major role in the establishment of pathological conditions associated with ROS imbalance, including diabetes mellitus [13–15].

In this review, we will focus our attention on the mechanisms regulating the correct balance and the complex interplay among ROS, NO, and p66^Shc^ that are crucial for EC function. We will also show how the alteration of this network is one of the driving pathogenetic mechanisms underpinning diabetic vasculopathy and endothelial dysfunction.

## 2. Endothelial Dysfunction in Diabetes Mellitus

### 2.1. NO Bioavailability Reduction and ROS Increase

**Figure 1:**
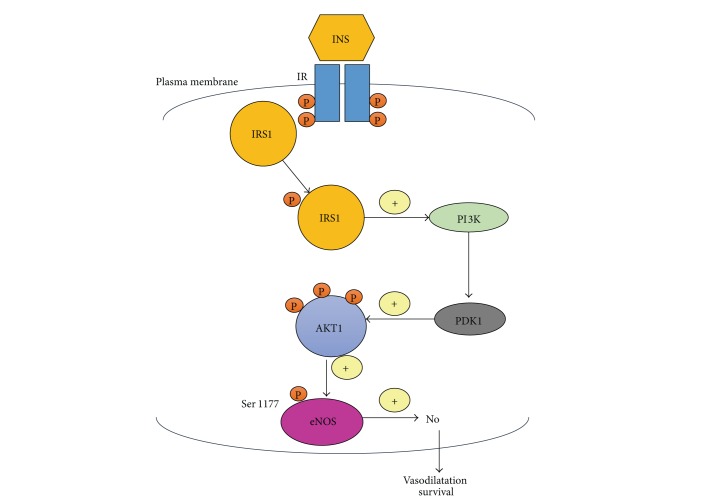
Intracellular insulin pathway. Insulin (INS), upon binding to its receptor, activates the insulin receptor tyrosine kinase, inducing tyrosine phosphorylation of the insulin receptor substrate-1 (IRS1). Phosphorylated IRS1 binds and activates phosphoinositol 3-kinase (PI3K), leading to the activation of serine-kinase phosphoinositide-dependent kinase 1 (PDK1), which activates AKT1. AKT1 phosphorylates eNOS at Ser-1177, leading to increased activity of eNOS and production of NO, which induces vasodilatation and EC survival. This pathway is strongly compromised in insulin resistance and diabetes mellitus.

The regulation of NO metabolism is particularly important in diabetes mellitus, since the activation of eNOS has been demonstrated to be under the insulin control [16–18]. In particular, it has been shown that insulin (INS) binding to its receptor activates the insulin receptor tyrosine kinase activity, resulting in tyrosine phosphorylation of the insulin receptor substrate-1 (IRS1). Phosphorylated IRS1 binds and activates phosphoinositol 3-kinase (PI3 K), leading to activation of serine-kinase phosphoinositide-dependent kinase 1 (PDK1), which phosphorylates and activates v-akt murine thymoma viral oncogene homolog 1 (AKT1). In turn, AKT1 directly phosphorylates eNOS at Ser-1177, leading to increased activity of eNOS and production of NO (Figure 1). Accordingly, IRS-1 mutations in ECs decrease insulin-stimulated eNOS phosphorylation and eNOS gene expression [19] and knockout mice of the endothelial-specific insulin receptor display decreased eNOS expression and endothelial vasodilator function impairment [20]. Moreover, animal models of insulin resistance, including the obese Zucker rat, display defects in the PI3 kinase/AKT1 system and impaired NO bioavailability [21].

Another signaling pathway that is activated by insulin in diabetes is the mitogen-activated protein kinases (MAPK) pathway viathe small GTPase Ras [22]. The Ras/MAPK insulin-signaling pathway generally leads to cellular growth and proliferation. In ECs, activation of this pathway has been linked to the expression of endothelin-1, which is a potent mitogen and vasoconstrictor, and to the expression of proinflammatory adhesion molecules such as ICAM-1 [23]. In diabetes mellitus and insulin resistance, insulin-mediated activation of eNOS viaPI3 kinase/AKT1 is inhibited, while the adverse effects of insulin remain unopposed, which may promote vascular disease [24]. Blood flow and other physiological stimuli activate eNOS viaPI3 kinase/AKT1; therefore, an impairment of this signaling mechanism in diabetes may have broad implications for vascular dysfunction.

NOX is a membrane associated multisubunit complex that generates superoxide anion and is involved in the oxidative burst of inflammatory cells as well as in EC signaling [25]. In pathological conditions, including diabetes mellitus, NOX activity and superoxide production are increased [26, 27]. Increased free fatty acid concentration activates NOX and the proinflammatory transcription factor NF*κ*B [28]. Moreover, NOX expression is upregulated by the systemic vasoconstrictor angiotensin II [29] that is increased in type 2 diabetic patients or animal models, along with its generating enzymes and receptors. Moreover, angiotensin II seems to play a role in the regulation of insulin secretion by pancreatic beta-cells and insulin sensitivity by peripheral tissues, which are two critical factors contributing to the development of type 2 diabetes. Accordingly, angiotensin converting enzymes inhibitors show positive vascular effects in diabetes [30, 31].

**Figure 2:**
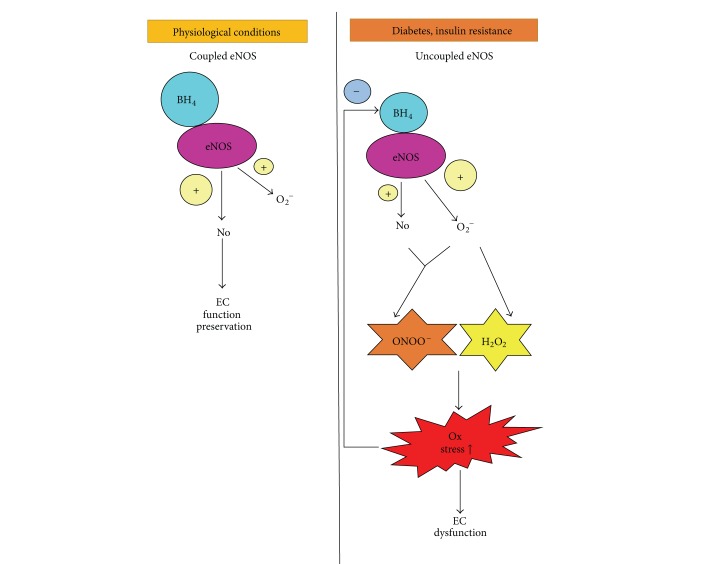
eNOS uncoupling in diabetes mellitus. Diabetes and insulin resistance are associated with eNOS uncoupling due to decreased levels of BH_4_. eNOS uncoupling leads to the production of superoxide anion (O_2_^−^), rather than NO. Superoxide, in turn, is dismutated to H_2_O_2_ or reacts with NO, leading to the formation of peroxynitrite (ONOO^−^) and to a further it decreases NO bioavailability. Increased ONOO^−^ and H_2_O_2_ levels induce oxidative stress that further aggravates BH_4_ depauperation.

Finally, diabetes is associated with eNOS uncoupling and decreased BH_4_ levels and therefore increased ROS production (Figure 2). In keeping with these data, BH_4_ supplementation improves NO production and endothelial function both in experimental diabetes models [32] and in human subjects with type 2 diabetes mellitus [33, 34].

Interestingly, Thomas et al. reported the effects of H_2_O_2_ treatment in ECs on eNOS phosphorylation. It was shown that a short H_2_O_2_ treatment with 300 *μ*M H_2_O_2_ induced promotion of eNOS activity and modulation of the eNOS phosphorylation status at Ser-1177 in porcine aortic endothelial cells via a calcium- and AKT-dependent pathway [35]. Additionally, another study demonstrated that eNOS mRNA expression was increased in bovine aortic ECs treated with 100 *μ*M H_2_O_2_ for 24 hours [36].

Most probably, both these effects might represent adaptive responses of ECs to maintain NO bioactivity under conditions of enhanced oxidative stress. On the other hand, a very interesting report demonstrated that human eNOS phosphorylation of Ser-1177, under conditions of eNOS uncoupling (i.e., in absence of BH_4_), increases the rate of superoxide anion generation instead of NO [37].

All the above studies demonstrate the intricate interplay existing between ROS and eNOS phosphorylation in Ser-1177 in relationship to BH_4_ levels (which are reduced under elevated levels of oxidative stress or diabetes; see Figure 2). Therefore, unbalanced red/ox homeostasis, as found in diabetes, will determine an exacerbated production of superoxide anion instead of NO, leading to diabetic-associated vascular dysfunction.

### 2.2. Oxidative Stress Insulin Resistance and Diabetic Complications

Oxidative stress plays a pivotal role in the development of insulin resistance and in both micro- and macro-vascular diabetic complications [38].

Moreover, diabetic condition is also associated with an impairment of cellular autophagy, a process involved in the degradation of cellular components [39]. Autophagy and oxidative stress are strictly related since autophagy is also responsible for organelles degradation, such as mitochondria, which are the sites of ROS production. Autophagy impairment, in fact, causes an accumulation of dysfunctional mitochondria leading to increased ROS production. Therefore the autophagy impairment associated with diabetes plays a causal role in ROS increase [39]. In diabetic conditions, in fact, there is a mitochondrial superoxide overproduction in ECs of both large and small vessels, as well as in the myocardium. Superoxide production is responsible for the activation of five major pathways involved in the pathogenesis of diabetes complications: polyol pathway flux, increased formation of advanced glycation end products (AGEs), increased expression of the AGE receptor and its activating ligands, activation of protein kinase C isoforms, and overactivity of the hexosamine pathway [38]. Moreover, it directly inactivates two critical antiatherosclerotic enzymes, eNOS (see paragraph above) and prostacyclin synthase. Through these pathways, increased intracellular ROS provokes angiogenesis impairment in response to ischemia, activates a number of proinflammatory pathways, and causes long-lasting epigenetic changes that drive persistent expression of proinflammatory genes after glycemia being normalized (hyperglycemic memory), described below in Section 5. Atherosclerosis and cardiomyopathy in type 2 diabetes are caused in part by pathway-selective insulin resistance, which increases mitochondrial ROS production from free fatty acids and by inactivation of antiatherosclerotic enzymes by ROS [38].Indeed, overexpression of superoxide dismutase in transgenic diabetic mice prevents diabetic retinopathy, nephropathy, and cardiomyopathy [38].

An important role in the establishment of insulin resistance is played by the redox sensitive transcription factor NF-E2-related factor 2 (Nrf2) [40]. Among the different mechanisms used by the cell to counteract sustained oxidative stress, Nrf2 regulates antioxidant response element (ARE/EpRE-) mediated expression of detoxifying and antioxidant enzymes and of the cystine/glutamate transporter involved in glutathione biosynthesis [41]. Nrf2/ARE activity decrease causes oxidative stress increase and mitochondrial dysfunction in the vasculature, leading to endothelial dysfunction, insulin resistance, and abnormal angiogenesis associated with diabetes [42]. Moreover it has been shown that the suppression of Nrf2 activity by the MAPK extracellular-signal-regulated kinase (ERK) is linked to the oxidative stress-induced insulin resistance in mice [43]. Since ERK is known to be a negative regulator of glucose uptake and to be responsible of oxidative stress-induced insulin resistance, a strong link does exist between these phenomena. In addition, Nrf2 expression in the heart has been shown to be first upregulated and then downregulated in late stages of diabetes in the mouse. In this contest, Nrf2 activation has been demonstrated both to suppress oxidative stress-induced ERK activity and to reverse oxidative stress-induced insulin resistance. Finally, antioxidants such as N-acetyl cysteine (NAC) and metallothionein can prevent oxidative stress-induced ERK activation and Nrf2 downregulation [43].

These results demonstrate that Nrf2 plays a critical role in regulating insulin sensitivity in the heart but presumably also in other tissue districts; therefore, targeting Nrf2 might provide a novel therapeutic approach for the treatment of insulin resistance and diabetic cardiomyopathy [43].

### 2.3. NO Bioavailability and Inflammation

Acute inflammatory states are known to impair endothelium-dependent vasodilatation since inflammatory mediators, such as tumor necrosis factor-*α* (TNF-*α*), decrease eNOS expression in ECs [44]. The inflammatory process induces the activation of ECs that is characterized by adhesion molecule expression, eNOS decrease, and consequent loss of NO bioactivity, all mechanisms that are critical for the promotion of the atherogenic phenotype [45]. Diabetes is associated with a systemic inflammatory state that may impair endothelial function and contribute to atherosclerosis [46]. Indeed, in diabetic patients there is an increase of circulating levels of inflammatory markers, including C reactive protein, TNF-*α*, and intercellular adhesion molecule-1 [47–49]. Moreover, higher levels of inflammatory markers are a predictor of increased cardiovascular risk in diabetic patients [50] and, on the other hand, augmented levels of circulating inflammatory markers also relate to the incidence of diabetes mellitus [51].

The nuclear factor *κ*B (NF*κ*B) transcription factor, a key regulator of inflammatory genes transcription, is also implicated in endothelial activation and is linked to the pathogenesis of insulin resistance [52]. Inflammatory mediators, such as inflammatory cytokines, free fatty acids, and the AGE receptor, activate NF*κ*B. They all induce the phosphorylation of the inhibitory subunit I*κ*B by the I*κ*B kinase (IKK-*β*), which allows the translocation of the regulatory subunits p50 and p65 to the nucleus, where they promote the expression of inflammatory genes. Indeed, overexpression of IKK-B or TNF-*α* in skeletal muscles causes insulin resistance [53]. Accordingly, *in vitro* and *in vivo* studies confirmed the existence of a link between insulin resistance and NF*κ*B activation, inflammation, and NO bioactivity impairment [54]. As a further confirmation, genetic suppression of IKK-*β* or pharmacological inhibition of IKK-*β* with a class of drugs named salicylates prevents insulin resistance. Accordingly, a large randomized trial was conducted in type 2 diabetes patients (TINSAL-T2D) testing an anti-inflammatory agent belonging to salicylate group, named salsalate (2-(2-hydroxybenzoyl) oxybenzoic acid). It was found that salsalate lowers hemoglobin A (1c) levels and improves glycemic control, showing the importance of chronic inflammation as pathogenetic mechanism of diabetes.

## 3. p66^Shc^ and Intracellular Oxidative Stress

In the oxidative stress-mediated responses, Shc proteins, intracellular adaptors regulating a variety of cellular functions, assume a major role [55]. There are three Shc mammalian genes: ShcA, ShcB, and ShcC. In mammals, ShcA is ubiquitously expressed, while ShcB and ShcC expression are limited to neuronal cells [56, 57].

The ShcA adaptor protein has three isoforms of 46, 52, and 66 kDa (p46^Shc^, p52^Shc^, and p66^Shc^, resp.) all generated from the same transcript, either through RNA splicing or alternative translational initiation [58, 59]. These three isoforms all share a common structure made of the CH_2_–PTB–CH_1_–SH_2_ modular domains [60]; the C terminal SH_2_ domain is necessary to bind to phosphotyrosine containing sequences; the PTB domain is a second domain capable of interacting with phosphorylated tyrosine residues independently; in particular, the p46^Shc^ isoform lacks the first 46 amino acids within the PTB domain. The CH_1_ domain lays between the PTB and SH_2_ domains and contains tyrosine residues that, upon phosphorylation, activate a specific signaling cascade. Finally, p66^Shc^ only has an additional domain at the N terminus, the CH_2_ domain. Of relevance, this domain contains a serine residue at position 36 (Ser-36) that is phosphorylated in response to several stress stimuli, including UV irradiation and H_2_O_2_ [61].

p52^Shc^ and p46^Shc^ proteins are inductors of the Ras signaling pathway [62–64]. In particular, p52^Shc^ is efficiently phosphorylated by the insulin receptor, causing the activation of the MAPK pathway that leads to cellular growth and proliferationviathe small GTPase Ras [22]. Interestingly, this pathway is not inhibited in diabetes mellitus and insulin resistance; conversely, the insulin-mediated activation of eNOS viaPI3 kinase/AKT1 is inhibited, limiting its vasodilator and prosurvival function and therefore promoting vascular disease [24].

The p66^Shc^ isoform function is not limited to signal transduction, since it is a redox enzyme implicated in mitochondrial ROS generation and translation of oxidative signals [55].

Under physiological conditions, the phosphorylation of Tyr residues of p66^Shc^ by growth factors mediates the signal transduction to the nucleus, inhibiting the Ras signaling pathway, while phosphorylation of the Ser-36 site seems to be crucial for oxidative stress response [65–67].

**Figure 3:**
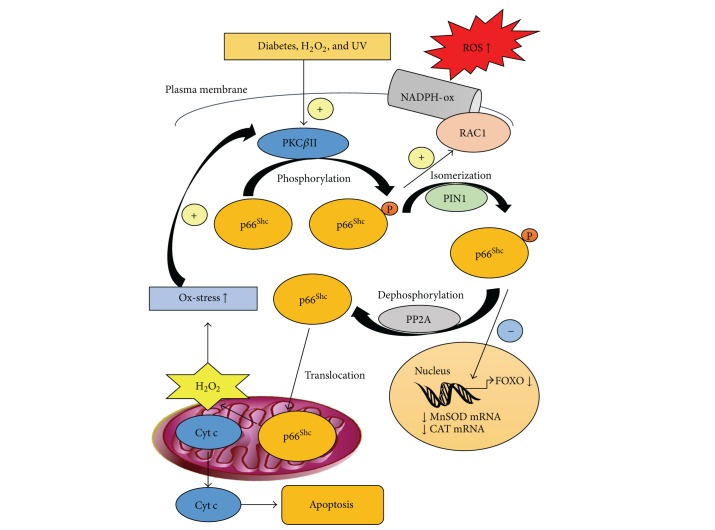
p66^Shc^ role in intracellular ROS production.  p66^Shc^ modulates ROS production by using three mechanisms. (1) The nuclear mechanism involves p66^Shc^ mediated FOXO transcription factors inhibition, leading to decreased expression of ROS-scavenging enzymes such as catalase (CAT) and manganese superoxide dismutase (MnSOD). (2) At the plasma membrane, p66^Shc^ promotes RAC1 activation and triggers NADPH membrane oxidase-ROS production. (3)  p66^Shc^ acts in the mitochondrial intermembrane space (IMS). In response to oxidative stress, p66^Shc^ is serine phosphorylated by PKC*β*II and isomerized by the peptidylprolyl cis/trans isomerase PIN1. This isomerization allows the dephosphorylation of Ser-36 residue by the serine threonine phosphatase PP2A, inducing the translocation from the cytosol to the IMS. In the IMS, p66^Shc^ binds to cytochrome c (Cyt c), generating ROS. These ROS activate the release of mitochondrial apoptotic factors, eventually inducing apoptosis.

p66^Shc^ modulates ROS production by using three mechanisms restricted in the nucleus, the plasma membrane, and the mitochondria, respectively. The nuclear mechanism involves p66^Shc^ mediated by forkhead box sub-group O** (**FOXO) transcription factors inhibition, leading to the decreased expression of ROS-scavenging enzymes CAT and manganese superoxide dismutase (MnSOD) [68]. At the plasma membrane, p66^Shc^ promotes RAC1 activation and triggers NADPH membrane oxidase-ROS production. A positive feedback loop between RAC1 and p66^Shc^ exists: RAC1, in fact, induces the phosphorylation-dependent increase of p66^Shc^ stability [69]. Finally, p66^Shc^ acts in the mitochondrial intermembrane space. In response to oxidative stress, p66^Shc^ is serine phosphorylated by protein kinase C*β*II (PKC*β*II) and isomerized by the peptidylprolyl cis/trans isomerase PIN-1 [70, 71]; this isomerization allows the dephosphorylation of Ser-36 residue by the serine threonine phosphatase PP2A, inducing the translocation from the cytosol to the mitochondrial intermembrane space, through the TIM/TOM mitochondrial import machinery. In the mitochondrial intermembrane space, p66^Shc^ binds to cytochrome c, acting as an oxidoreductase and generating ROS. These ROS, in turn, activate the permeability transition pore, triggering organelle dysfunction, massive release of mitochondrial apoptotic factors, and ROS and eventually inducing cell apoptosis [71] (Figure 3).

Indeed, increased p66^Shc^ content in the mitochondria correlates with alteration of mitochondrial structure, decrease of mitochondrial calcium uptake, and enhanced mitochondrial ROS production, triggering the mitochondrial route of apoptosis [70]. It is worth noting that the mitochondrial H_2_O_2_ production induced by p66^Shc^ further increases intracellular H_2_O_2_ levels, maintaining or increasing PKC*β*II activation in a positive control loop [72, 73].

In keeping with its proapoptotic function, p66^Shc^ can be also phosphorylated by apoptosis signal-regulating kinase 1 (ASK1) [74] and p38 MAPK [75]. Indeed, p38 MAPK is target of several factors inducing ROS generation, including osmotic and thermic shock, inflammatory cytokines, lipopolysaccharides, ultraviolet light, interleukin 1, and H_2_O_2_ [75].

In agreement with these data, increased resistance to oxidative stress has been observed in p66^Shc^ knockout mice (p66^Shc−/−^) [61] and characterized by reduced oxidative stress-induced apoptosis, prolonged lifespan, reduced production of intracellular oxidants, and increased resistance to oxidative stress-induced apoptosis, which is restored by p66^Shc^ overexpression [61]. Consistently, p66^Shc−/−^ mice show reduced levels of systemic (isoprostane) and tissue (nitrotyrosine, 8-oxo-dG) oxidative stress markers [68, 76, 77] and enhanced resistance to oxidative stress induced by hypercholesterolemia [78], angiotensin II [79], carbon tetrachloride, and ethanol [80]. Indeed, our group demonstrated that p66^Shc^ deletion increased both skeletal muscle and EC resistance to ischemia [81]. Intriguingly, we also found that p66^Shc^ not only inhibited cell survival but also differentiation of skeletal muscle progenitors and skeletal muscle regeneration after hindlimb ischemia [82].

These observations seem to be of clinical relevance. Indeed, p66^Shc^ mRNAs is increased in peripheral blood monocytes of patients with acute coronary syndrome, but not in those who display stable coronary artery disease [83]. In addition, plasma levels of malondialdehyde, an established marker of lipid peroxidation and thus of oxidative stress, correlate positively with p66^Shc^ expression.

### 3.1. p66^Shc^, ROS and Diabetes Mellitus

Different reports unravel a major role of p66^Shc^ also in response to hyperglycemia (HG) and diabetes conditions that both upregulate oxidative stress.

There are several studies exploring the interactions between hyperglycemia-associated ROS production and p66^Shc^ in diabetes animal models. Indeed, streptozotocin (STZ) treated mice, a model of type 1 diabetes, show higher expression of p66^Shc^ compared to nondiabetic mice. In addition, STZ treated p66^Shc−/−^ and wild type (wt) mice show a similar increase in blood glucose but significant differences with respect to endothelial dysfunction and oxidative stress production [84]. Peroxynitrites are formed after the reaction between free radicals, such as superoxide and NO, decreasing NO availability and enhancing cellular oxidative stress and eventually leading to endothelial dysfunction [85]. In accordance with the decreased oxidative stress levels observed upon diabetes induction, p66^Shc−/−^ mice also display reduced peroxynitrite production, and thus contributing to blood vessel relaxation [84].

Increased glucose levels impact directly on p66^Shc^. Indeed, HG-mediated ROS production induces phosphorylation of the Ser-36 residue of p66^Shc^, leading to the collapse of mitochondrial transmembrane potential [86].

A well-known effect of HG is the formation and accumulation of AGEs, which can further amplify oxidative damage by increasing oxidative stress in cells [87, 88]. Ser-36 p66^Shc^ phosphorylation mediated through AGE-induced ROS production has been shown to be responsible for FOXO3A inhibition [89].

Additionally,HG-mediated ROS overproduction also activates AKT1/PKB kinase, which phosphorylates and inactivates FOXO3A protein, inducing oxidative stress and depressing the survival phenotype [90]. This is of particular importance for diabetes-associated redox imbalance, since FOXO3A transcription factor is responsible for SOD2 and catalase ROS scavenger synthesis [91–93]. p66^Shc^-AKT interplay also affects NO production: p66^Shc^ silencing leads to activation of Ras and AKT kinase, with a corresponding increase in phosphorylation of eNOS at Ser-1177. Accordingly, in rat aortic rings, knockdown of p66^Shc^ suppresses the vasoconstrictor responses enhancing vasodilatation [94].

Another signaling pathway altered in HG involves NF*κ*B and NOX4 (NADPH oxidase 4),that are elevated in p66^Shc^ wt but not in knockout animals [95].

Finally, p66^Shc^ is essential also for glucose uptake in skeletal muscle cells. In fact, p66^Shc^ protein regulates MAPK activity and the actin cytoskeleton turnover [96], which are both required for normal glucose transport regulation. Loss of p66^Shc^ in rat myoblasts activates MAPK activity, leading to altered cell cytoskeleton and resulting in strong increase in basal glucose transport [97]. Moreover glucose transporter proteins GLUT1 and GLUT3 are induced too. On the other hand, in rat myoblasts overexpressing p66^Shc^, basal glucose uptake rate is significantly reduced and the cellular levels of glucose transporters GLUT1 and GLUT3 are decreased. Thus, p66^Shc^ may represent an effector of glucose transport in skeletal muscle cells and confirm to play an important role in the adaptive responses to environmental factors [97].

Again, these observations appear to be of clinical relevance. Indeed, p66^Shc^ mRNA level is significantly increased in mononuclear blood cells from type 2 diabetic patients compared to healthy controls and it correlates positively with total plasma isoprostanes, well-known markers of oxidative stress [98].

#### 3.1.1. p66^Shc^ and Endothelial Progenitor Cells (EPCs) Function in Diabetes Mellitus

EPC levels in diabetic patients are significantly reduced compared with control subjects. EPCs derived from diabetic patients, in fact, produce excessive ROS and show impaired migratory capacity [99]. eNOS uncoupling explains, at least in part, the reduced levels and impaired function of EPCs observed in diabetes contributing to the pathogenesis of vascular disease [99].

Accordingly, mouse bone marrow derived c-kit^+^ cells of p66^Shc−/−^ mice are resistant to both apoptosis and oxidative stress induced by high glucose [100]. Upon oxidative stress, the bioavailability of NO is reduced, and consequently endothelial function and differentiation are impaired. HG resistance conferred by p66^Shc^ deletion is dependent on the activity of NOS and, accordingly, a NO donor is sufficient to rescue bone marrow-derived EPCs deficit induced by HG [100]. In line with *in vitro* data, the knockout of p66^Shc^ prevents the diabetic impairment of capillary network formation in a mouse model of angiogenesis [100], strongly indicating that p66^Shc^ represents a promising therapeutic target for the prevention, the development, and the progression of diabetic vasculopathy.

### 3.2. p66^Shc^ Role in Hypercholesterolemia

Hypercholesterolemia increases ROS and reactive nitrogen species production, resulting in oxidation and peroxidation of lipids, proteins, and lipoproteins [101–104]. In p66^Shc−/−^ mice chronically fed with a high-fat diet, the levels of oxidized low-density lipoprotein (LDLs) and of isoprostanes, produced from polyunsaturated fatty acids through radical-catalyzed mechanisms, are reduced as well as the formation of intimal macrophage-derived foam cells in the arterial wall. Thus, loss of p66^Shc^ expression protects against oxidative stress and early lesion formation [78]. Furthermore, in p66^Shc−/−^ mice, low atherogenesis and reduced oxidative stress are coupled with reduced apoptosis in aortic lesions [78].

In an interesting study, the relationships among lipids, oxidative stress, and p66^Shc^ were investigated in peripheral white blood cells and in subcutaneous adipose specimens of patients displaying either high or low LDL plasma levels [105]. It was reported that p66^Shc^ mRNA levels in WBC and in adipose tissue were directly related to LDL expression, and multiple regression analysis showed that LDL plasma levels were the only variable affecting p66^Shc^ mRNA expression [105].

### 3.3. p66^Shc^ Role in Aging

Aging is an independent risk factor for cardiovascular diseases and senescent vascular cells are present in human atherosclerotic tissues [106], suggesting that vascular cell senescence could be linked to the pathophysiology of age-related vascular diseases. Accordingly, vascular cell senescence has been also shown in diabetic vasculopathy [107, 108]. Indeed, HG-induced endothelial senescence leads to vascular inflammation and thrombosis, exacerbating the diabetic-associated cardiovascular events.

Calorie restriction in mammals decreases the incidence of age-associated disorders including cardiovascular diseases [109]. “Silent mating type information regulation 2 homolog” (sirtuin 1 or SIRT1) is NAD^+^-dependent class III histone deacetylase (HDAC) found up-regulated under caloric restriction and extending the lifespan of many organisms [110]. A SIRT1 upregulation and/or activation is associated with EC functional preservation; whereas excessive ROS or aging decrease SIRT1 expression, leading to endothelial dysfunction [111]. In fact, SIRT1 activation in ECs ameliorates oxidative stress response, prevents endothelial senescence, and promotes eNOS-derived NO bioavailability and mitochondrial biogenesis [112–114].

SIRT1 has been shown to target p53, FOXO3, and eNOS for deacetylation, negatively regulating oxidative stress [114–116]. Recently, it has been demonstrated that the endothelium-specific overexpression of SIRT1 is able to inhibit the HG-induced upregulation of the senescence-associated markers, such as the CDK inhibitor p21^Waf1/Cip1/Sdi1^ (p21), p53, and the plasminogen activator inhibitor-1 (PAI-1) [117]. Furthermore, SIRT1-transgenic diabetic mice exhibited decreased expression of p66^Shc^ and increased expression of the scavenging enzyme MnSOD [117]. Indeed, SIRT1 has been shown to repress p66^Shc^ transcription at the chromatin level: SIRT1 overexpression decreased acetylated histone H3 binding to the p66^Shc^ promoter region; whereas inhibition of SIRT1 increased acetylated histone H3 binding to the same region. Therefore, the decreased levels of p66^Shc^ attributable to SIRT1 could be the result of a direct inhibitory role of SIRT1 on p66^Shc^ expression through epigenetic chromatin modifications [112].

Overall, these data suggest that the protective role of SIRT1 against hyperglycemia-induced vascular cell senescence is mediated, at least in part, through the reduction of oxidative stress through a cross-talk with p66^Shc^.

The above described role of p66^Shc^ in endothelial dysfunction associated with different disease conditions is summarized in Table 1.

## 4. MicroRNAs Oxidative Stress and Diabetes

MicroRNAs (miRNAs) are small noncoding RNAs that regulate stability and translational inhibition of target messenger RNAs (mRNAs). miRNAs are involved in most biological processes, including proliferation, differentiation, development, migration, and apoptosis [11, 12]. miRNA dysregulation has been observed in the development of different diseases, including diabetes mellitus [13–15]. Specifically, several miRNAs modulated by oxidative stress have been demonstrated to be dysregulated in diabetes and to cause vasculopathy (Table 2).

Among them, miR-200 family members have been shown to play a causative role in the establishment of vascular diabetic inflammatory phenotype [118]. This miRNA family consists of five members, miR-200c, miR-141, miR-200a, miR-200b, and miR-429 and it has been widely studied for its role in the epithelial to mesenchymal transition of tumor cells [119].

We found that miR-200 family is induced upon H_2_O_2_ treatment in ECs and in particular one of its members, miR-200c, is responsible for the induction of apoptosis and senescence [120]. This is likely physiologically relevant since miR-200 family induction is also observed in a mouse model of hindlimb ischemia with an oxidative stress mediated mechanism [81]. Indeed, miR-200c and miR-200b upregulation is markedly inhibited in ischemic p66^Shc−/−^ mice, which display lower levels of oxidative stress in basal conditions [10] and after ischemia [81], supporting a role of ROS in miR-200 family induction [120].

An upregulation of miR-200 family members has been also reported in VSMCs of diabetic mice (db/db) compared to control db/+ mice [118]. In particular, the authors found that miR-200b, miR-200c, and miR-429 expression levels were increased in VSMCs of diabetic mice, while the protein levels of their common target ZEB1 were decreased. Overexpression of miR-200 mimics the downregulated transcriptional repressor ZEB1, leading to the transcription of the inflammatory genes cyclooxygenase-2 (COX-2) and monocyte chemoattractant protein-1 (MCP-1) that in turn promote monocyte binding to VSMCs, eliciting a proinflammatory response. In accordance with these results, ZEB1 occupancy of inflammatory gene promoters was reduced in db/db VSMCs [118].

Notably, miR-200 family is also induced by NO [121]. NO treatment and miR-200-family overexpression, or ZEB2 knockdown, all elicit the expression of mesendoderm and early cardiovascular precursor markers, including fetal liver kinase 1 (Flk1) and chemokine receptor type 4 (CXCR4), inducing mouse embryonic stem (ES) cells differentiation towards the mesoendoderm and cardiovascular lineage [121].

Another miRNA that is found significantly upregulated in VSMCs of diabetic mice (db/db) compared to control mice db/+ is miR-125b [122] that is also induced by oxidative stress in human keratinocytes HaCaT exposed to H2O2 [123]. miR-125b protein target is the histone H3 lysine-9 methyltransferase Suv39 h1. In diabetic VSMCs, there is a decreased promoter occupancy of Suv39 h1 in inflammatory genes and, consequently, of the associated repressive epigenetic mark histone H3 lysine-9 trimethylation (H3K9me3) [122]. This study supports the idea that epigenetic mechanisms implicated in the upregulation of inflammatory genes in ECs and VSMCs under diabetic conditions, at least in part, pass through oxidative stress-dependent modification of miRNA expression (see section below).

Recently, it has been shown that decreased levels of miR-27b are present in bone marrow-derived angiogenic cells (BMACs) from both type 2 diabetes mellitus patients and type 2 diabetic db/db mice [124]. miR-27b under normoglycemic condition protects BMAC angiogenesis, represses mitochondrial reactive oxygen species, and improves wound healing by targeting the antiangiogenic molecules semaphorin 6A (Sema6A), p66^Shc^, and thrombospondin-1 (TSP-1), respectively [124]. In contrast, in diabetes mellitus, miR-27b expression is decreased, which harms BMAC angiogenesis and increases mitochondrial ROS production [124]. Overexpression of miR-27b, in fact, rescues BMAC functions and improves BMAC therapy on diabetic wounds, accelerating wound closure and increasing wound perfusion. These data indicate that miR-27b gene therapy enhances the efficacy of diabetic angiogenic cells for wound angiogenesis and wound repair in diabetic subjects [124].

Another miRNA that plays a major role in EC function is miR-210 [125]. In particular, its upregulation stimulates EC angiogenesis, at least in part, through the downmodulation of Ephrin A3 (EFNA3) [125]. Moreover, miR-210 is involved in mitochondrial ROS production targeting many mitochondrial components [3] and miR-210 blockade decreases EC survival upon ischemia with an oxidative stress mediated mechanism [126]. Interestingly, miR-210 levels are also induced in the failing heart of postischemic type 2 diabetic patients [127]. Thus, while miR-210 levels have not been measured in cardiac endothelium specifically, dysregulation of this miRNA is likely to play a role in diabetic EC dysfunction.

## 5. Epigenetic Modulations Induced by ROS in Diabetes Mellitus

As previously described, epigenetic modulations elicited under diabetic conditions have been proven to play a pivotal role in the progression of the disease and, importantly, in hyperglycemic memory. The latter likely underpins the failure of intensive glucose control in the improvement of cardiovascular outcomes in diabetic patients [9].

*In vitro* studies demonstrate that epigenetic mechanisms modulate glucose-induced gene expression of the subunit p65 of NF*κ*B. This phenomenon has been assigned to the activation of SET7/9 histone methyltransferase which methylates lysine 4 on histone H3 and promotes gene transcription [128]. The overall process leads to NF*κ*B activation and to the expression of proinflammatory molecules such as VCAM-1 and MCP-1.

The emerging concept is that epigenetic modifications are at the basis of high glucose-dependent modifications and may be involved in the onset of hyperglycemic memory. Although this observation still awaits confirmation in humans, it is certainly intriguing and worth of further investigations. A possible problem could be that not all the cells in the body may be equally sensitive to high glucose, resulting in a persistent modification of gene expression and/or cell function. Our knowledge in this direction is still limited and further analyses are required to understand the molecular basis of hyperglycemic/epigenetic memory as well as the potential interventions we may design to correct the problem [129]. Intriguingly, the same proinflammatory genes are induced in models of diabetes-associated atherosclerosis, suggesting that similar mechanistic processes may underlie different physiopathological outcomes associated with the same metabolic alteration [130, 131].

Evidence is emerging that ROS could be key mediators underpinning glucose mediated epigenetic modulations; in particular, mitochondrial ROS have been shown to be implicated in the epigenetic changes that induces NF*κ*B activation [129, 132]. Recently, a study shows that. in ECs exposed to high glucose and in aortas of diabetic mice, activation of p66^Shc^ by PKC*β*II continues after returning to normoglycemia [133]. This persistent p66^Shc^ upregulation and mitochondrial translocation is associated with continued ROS production, apoptosis, and reduced NO bioavailability. In particular, p66^Shc^ gene is epigenetically regulated by its promoter CpG hypomethylation and by GCN 5-induced histone 3 acetylation, causing p66^Shc^ overexpression [133]. Accordingly, also other oxidative stress inducing stimuli seem to act with a similar mechanism: cholesterol upregulates EC p66^Shc^ expression via hypomethylation of two CpG dinucleotides and acetylation of histone 3 in its promoter [134].

Furthermore, p66^Shc^-derived ROS production induces PKC*β*II upregulation that, in turn, phosphorylates and inhibits eNOS, leading to a damaging vicious cycle even after restoration of normoglycemia*. In vivo* and *in vitro* gene silencing of p66^Shc^ after glucose normalization restores endothelium-dependent vasorelaxation and, decreasing ROS production, inhibits apoptosis [133].

The above described studies underline the importance of the relationship occurring between p66^Shc^, ROS production, eNOS activity, and inflammation that all concur to the establishment of the hyperglycemic memory and of vascular diseases associated with diabetes.

## 6. Clinical Use of Antioxidants to Improve Endothelial Function

Given the fundamental role of oxidative stress in the onset of diabetes and diabetic complications, several attempts to target redox imbalance pharmacologically have been conducted [135].

A number of interventional trials were conducted between 1996 and 2002, testing vitamin E, *β*-carotene, and vitamin C, alone or in combination, and at different dosages. Although some studies showed a benefit of vitamin E administration in the secondary prevention of cardiovascular disease [136] and of vitamin E + C supplementation in slowing carotid intima-media thickening in hypercholesterolaemic patients [137], clinical trials gave heterogeneous outcomes. Therefore, in 2004, the American Heart Association Committee for Nutrition, Physical Activity, and Metabolism discouraged the use of antioxidant for cardiovascular disease prevention [138].

The neutral effect of vitamin E + C administration was also confirmed by the results of the 2008 Physicians Health Study that enrolled more than 14000 middle-aged male with low prevalent cardiovascular disease and followed them up for about 10 years [139]. Importantly, questions have been raised about the safety of antioxidant vitamins, since an increased overall mortality associated with *β*-carotene, vitamin E, and vitamin A supplementation was reported in some of these trials, possibly due to increased cancer mortality [140]. In conclusion, these interventional trials did not confirm that pharmacological correction of the redox status with antioxidant vitamins could be used as a safe and effective therapeutic strategy.

However, another group of antioxidant compounds, polyphenols, comprising about 8000 different molecules, among which flavonoids are the most studied, holds good promise. Polyphenols are abundant in vegetables and particularly in products such as wine, chocolate, and tea. The antioxidant capacity of these compounds is attributable both to the inhibition of enzymatic sources of ROS and to the stimulation of antioxidant mechanisms [141]. Indeed, a meta-analysis of 113 studies testing different food, beverages, or extracts supplementations demonstrated that polyphenols improve endothelial function, both in healthy subjects and in patients with cardiovascular risk factors, reducing blood pressure [142].

One of the most studied polyphenols is resveratrol that is contained in red grapes as well as in other fruits. Animal studies demonstrated that resveratrol displays a strong antidiabetic effect, decreasing blood glucose in hyperglycemic rodents [143]. This effect seems to result from increased intracellular transport of glucose. Moreover, resveratrol was also demonstrated to have effects that may contribute to the protection of *β*-cells in diabetes. Indeed, in rat pancreatic islets experiments, resveratrol was shown to reduce insulin secretion [143] and this event was confirmed in rats with hyperinsulinemia, in which resveratrol decreased blood insulin levels [144]. Moreover, resveratrol inhibited cytokine action and attenuated the oxidative damage of the pancreatic tissue [145]. Additionally, studies in animal models of insulin resistance indicate that resveratrol increases insulin function (reviewed in [144]). The improvement of insulin action mechanism is complex and involves reduced adiposity and changes in gene expression and in the activities of different enzymes. Finally, resveratrol has been demonstrated to increase Nrf2 activity, supporting its antioxidant effect [146] and to be a potent inducer of SIRT1, a key molecule that regulates energy homeostasis, mitochondrial biogenesis, and insulin sensitivity, as above described [147].

In spite of the encouraging preclinical data, clinical intervention studies performed so far show conflicting effects of resveratrol between trials (reviewed in [148]). Some trials revealed an insulin sensitivity increase and glucose control, whereas others did not report positive effects [148]. Factors that may influence the outcome of the trials are resveratrol doses and the timing of consumption as well as the metabolic status of the subject.

Another approach aimed at modulating oxidative stress in humans takes advantage of drugs directed primarily to other targets. Angiotensin-receptor blockers and ACE inhibitors have been found to exert nonhemodynamic beneficial effects on endothelial function by inhibiting NOX activity, reducing subunits expression and vascular oxidative stress [149]. Similarly, statins reduce NOX-mediated ROS production and activate NOS function [150]. After a short time of treatment with statins, an improvement in the endothelial function of the forearm vasculature was demonstrated, prior to any lipid-reducing effect [151]. In summary, the beneficial effects obtained by a therapy with ACE inhibitors and/or statins seem to be, at least in part, mediated by properties that are independent of the hemodynamic or cholesterol-lowering effects of these drugs.

Another approach to prevent ROS generation is inhibiting the enzyme system xanthine-oxidase, a well-known endothelial and cardiac source of superoxide [152]. Indeed, in one trial, the inhibition of xanthine-oxidase with allopurinol was shown to improve endothelial dysfunction in patients with type 2 diabetes with mild hypertension [153].

Several explanations can be envisioned to explain the largely conflicting results obtained so far in interventional trials testing antioxidant strategies. It must be underlined that ROS play a fundamental role as second messengers in cell physiology and that in low concentrations some ROS, particularly H_2_O_2_, are very important for cell growth and angiogenesis; moreover, a crucial role of ROS has also been shown in protective mechanisms such as preconditioning [154]. Therefore, the administration of antioxidants that is based on the concept that these molecules only have damaging effects should be reconsidered since, in certain settings, ROS quenching might have deleterious implications that counterbalance the positive ones. Methodological issues also need to be carefully considered: for instance, dosage, treatment duration, choice of outcome measures, populations under study, and concomitant therapy [155].

Collectively, these considerations provide a sufficient explanation for the failure of antioxidants tested so far. Evidence indicates that we need more hypothesis-driven and rigorous clinical trial designs, guided by a better understanding of the complex physiopathological role of ROS. Future research will have to develop newer antioxidant molecules, more specific, with a better pharmacodynamic profile or ancillary effects or impacting systemic and tissue oxidative stress through different mechanisms.

## 7. Conclusions

We described in this review how oxidative stress, modulating p66^Shc^-NO pathway, impinges in many crucial aspects of diabetic endothelial dysfunction, representing a promising therapeutic objective. So far, however, it is still unclear which pathogenetic mechanism should be targeted and the class of drugs that may be useful. In our vision, a more accurate knowledge of the fundamental disease mechanisms is the only way to the identification of a therapeutic strategy targeting oxidative stress.

## Figures and Tables

**Table 1:** p66^Shc^ knockout mice phenotypes.

Disease	Phenotype	p66^Shc+/+ ^	p66^Shc−/−^
Diabetes	Peroxynitrite production	+++	+
	Lipid peroxidation	+++	+
	p66^Shc^ expression	++	−

Aging	NO availability	+	+++
	O_2_ production	+++	+
	Protein nitration	+++	+
	iNOS expression	+++	+

Hypercholesterolemia	Aortic lesions area	+++	+
	Plasmatic isoprostanes	+++	+
	Lipid peroxidation	+++	+
	Vascular apoptosis	+++	+

+++: high expression or production; ++: medium expression or production; +: moderate expression or production; −: no expression.

**Table 2:** Modulated miRNAs in diabetic endothelial dysfunction and oxidative stress.

ROS source/disease	miRNAs	Tissue/organ	Source	Target	Functions	References
	Upregulated					
H_2_O_2_	miR-200c, miR-141, miR-200a, miR-200b, and miR-429	ECs, myoblasts	Human	ZEB1	Apoptosis, senescence	[120]
Diabetes	miR-200c, miR-200b, and miR-429	VSMCs	Mouse	ZEB1	Inflammation	[118]
NO	miR-200c, miR-200a, miR-200b, and miR-429	mES	Mouse	ZEB2	Mesendoderm and cardiovascular differentiation	[121]
Hypoxia	miR-210	ECs	Human	EFNA3	Angiogenesis	[125]
Diabetes	miR-210	Failing heart	Human			[127]
Diabetes	miR-125	VSMCs	Mouse	Suv39h1	Inflammation	[122]
	Downregulated					
Diabetes	miR-27b	BMACs	Human/mouse	Sema6A, p66^shc^, and TSP-1	ROS production; angiogenesis impairment	[124]

BMACs: bone marrow-derived angiogenic cells, ECs: endothelial cells, EFNA3: Ephrin A3, H_2_O_2_: hydrogen peroxide, mES: mouse embryonic stem, NO: nitric oxide, p66^Shc^: p66 isoform of ShcA protein, ROS: reactive oxygen species, Sema6A: semaphorin 6A, Suv39h1: suppressor of variegation 3-9 homolog 1 (Drosophila), TSP-1: thrombospondin-1, VSMCs: vascular smooth muscle cells, ZEB1: zinc finger E-box binding homeobox 1, and ZEB2: zinc finger E-box binding homeobox 2.
